# Beyond ‘warm and wet’: refining congestion phenotypes within the Nohria–Stevenson framework in acute heart failure

**DOI:** 10.1093/eschf/xvag226

**Published:** 2026-08-27

**Authors:** Rebecca Urtiga, Luis Fernando Boff Zarpelon

**Affiliations:** Universidade Federal da Integração Latino-Americana, Avenida Tarquínio Joslin dos Santos, 1000, Foz do Iguaçu, Paraná, Brazil Curso de Medicina, Centro Interdisciplinar de Ciências da Vida, Instituto Latino-Americano de Ciências da Vida e da Natureza; Universidade Federal da Integração Latino-Americana, Avenida Tarquínio Joslin dos Santos, 1000, Foz do Iguaçu, Paraná, Brazil Curso de Medicina, Centro Interdisciplinar de Ciências da Vida, Instituto Latino-Americano de Ciências da Vida e da Natureza

**Keywords:** Heart Failure, Haemodynamics, Patient classification, Point-of-Care Ultrasound, Congestion

Clinical classifications are useful only when they help clinicians make better decisions. In acute heart failure (AHF), the first bedside question is whether the patient is congested, hypoperfused, both, or neither. The Nohria–Stevenson warm/cold and wet/dry profiles remain attractive because they are simple, memorable, and closely aligned with initial management.^1^ The invasive Forrester subsets were defined by pulmonary capillary wedge pressure and cardiac index in acute myocardial infarction,^2^ whereas the Nohria–Stevenson profiles translated analogous logic into bedside signs in hospitalized heart failure.^1^ Contemporary guidelines also emphasize early recognition of congestion, hypoperfusion, pulmonary oedema, right-sided heart failure, and shock as clinically actionable phenotypes.^3,4^ The present Viewpoint preserves this logic and proposes a narrower refinement: patients who are wet may be described by the dominant congestion phenotype at presentation.

The warm-and-dry profile remains an essential part of bedside assessment. Patients with known or suspected heart failure commonly present with dyspnoea caused by chronic lung disease, infection, anaemia, deconditioning, arrhythmia, angina, or compensated chronic heart failure. In these situations, preserved perfusion without objective congestion is diagnostically valuable because it argues against acute heart failure as the dominant explanation for symptoms and should prompt reassessment for alternative or concomitant causes rather than reflex escalation of intravenous diuretics, vasoactive drugs, or high-acuity monitoring.

The original Nohria *et al.* cohort supports this broad diagnostic use. Among 452 hospitalized patients referred to a cardiomyopathy service with a diagnosis of heart failure, only 49% were admitted primarily for decompensated heart failure; others were admitted for arrhythmia (12%), angina (12%), elective transplant evaluation for chronic heart failure (17%), or other conditions.^1^ This heterogeneity helps explain why warm-and-dry patients were common and highlights the distinction between elective or broad heart failure evaluation and acute-care decisions in emergency or hospital settings.

The Nohria–Stevenson framework can be preserved while improving the clinical resolution of congested presentations. A practical approach is to retain the four traditional bedside profiles and add a descriptive layer within the wet spectrum (*Figure 1*). In this approach, any objective pulmonary and/or systemic congestion remains wet. Among warm-and-wet patients, the dominant pattern can be described as Warm/Wet-R when redistribution-predominant pulmonary congestion is the leading presentation, or Warm/Wet-V when volume-overload-predominant systemic or mixed congestion is predominant. The same qualifier may be applied descriptively to cold-and-wet patients, although hypoperfusion remains the dominant risk signal and therapeutic priority in that setting.

**Figure 1:**
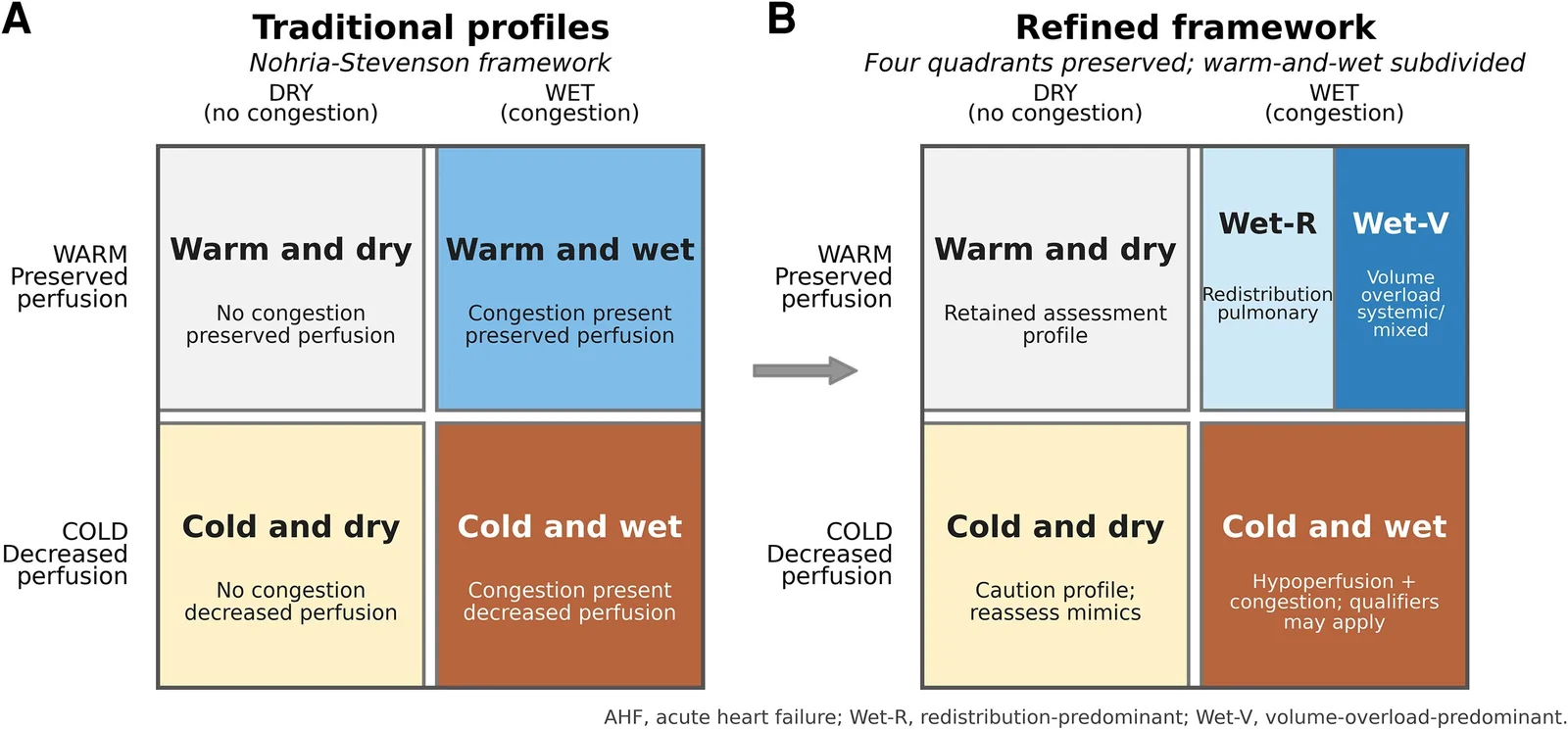
Refinement of congestion phenotypes within the Nohria–Stevenson bedside haemodynamic framework. *Panel A* shows the traditional Nohria–Stevenson warm/cold and wet/dry profiles. *Panel B* preserves the original four-profile structure while subdividing the warm-and-wet quadrant into Warm/Wet-R, indicating redistribution-predominant pulmonary congestion, and Warm/Wet-V, indicating volume-overload-predominant systemic or mixed congestion. Warm-and-dry remains a clinically useful assessment profile, whereas Wet-R/Wet-V qualifiers may also be applied descriptively to cold-and-wet patients when appropriate. AHF, acute heart failure

Warm/Wet-R identifies a wet presentation dominated by pulmonary congestion and redistribution physiology. This phenotype is often characterized by abrupt symptom onset, marked respiratory distress, orthopnoea, diffuse B-lines or radiographic pulmonary oedema, severe hypertension or acute afterload mismatch, and absent or limited systemic venous congestion. Its immediate management may prioritize oxygenation, ventilatory support when needed, treatment of the trigger, and afterload reduction when blood pressure allows, while the intensity of decongestion should be individualized and reassessed early.

Warm/Wet-V identifies a wet presentation dominated by systemic or mixed congestion and volume-overload physiology, with or without pulmonary involvement. Typical features include elevated jugular venous pressure, peripheral oedema, ascites, hepatomegaly, hepatojugular reflux, pleural effusions, renal or hepatic venous congestion, and progressive fluid accumulation. This profile corresponds to the dominant congestive presentation in acute heart failure registries.^5,6^ It is not a simple left-sided vs right-sided distinction. Many patients have biventricular disease, and pulmonary and systemic congestion frequently coexist.

This subdivision may be useful for three reasons. First, mechanisms may differ, such as abrupt pressure redistribution during hypertensive pulmonary oedema versus progressive salt and water accumulation in chronic heart failure. Second, bedside presentations may differ, with respiratory distress predominating in one patient and systemic venous congestion predominating in another. Third, immediate stabilization priorities may differ: redistribution-predominant presentations may require early non-invasive or invasive ventilatory support and afterload reduction, whereas volume-overload-predominant presentations usually place greater emphasis on sustained decongestion and monitoring of diuretic response.

Operationally, these categories should be considered predominant phenotypes rather than mutually exclusive compartments. Pulmonary congestion may be supported by orthopnoea, crackles, radiographic oedema, elevated natriuretic peptides, and lung ultrasound; three or more B-lines in two or more bilateral zones supports congestion in the appropriate context.^7^ BNP ≥100 pg/mL or age-adjusted NT-proBNP thresholds of ≥450, ≥900, and ≥1800 pg/mL for patients aged <50, 50–75, and >75 years, respectively, support AHF when the clinical context is compatible.^8^ Systemic congestion is supported by elevated jugular venous pressure, oedema, ascites, positive hepatojugular reflux, pleural effusions, inferior vena cava plethora, or venous point-of-care ultrasound; where available, VExUS integrates inferior vena cava size with hepatic, portal, and intrarenal venous Doppler patterns.^9^ Hypoperfusion should not be inferred from blood pressure alone, but from a pattern including cool extremities, narrow pulse pressure, altered mentation, oliguria, or elevated lactate.^3,4,10^

Mixed presentations and conflicting findings should be expected. Patients with hypertensive pulmonary oedema may have mildly elevated jugular venous pressure, and chronically volume-overloaded patients may have B-lines that improve rapidly. When both compartments are involved, classification should reflect the dominant clinical presentation and the primary therapeutic priority at that assessment. If uncertainty persists, the safest approach is early reassessment after initial stabilization and response to therapy.

The therapeutic implications are pragmatic rather than prescriptive. The Wet-R versus Wet-V distinction does not mandate fixed diuretic doses. Diuretic intensity should remain individualized according to prior loop diuretic exposure, blood pressure, renal function, urine output or natriuretic response when available, degree of systemic venous congestion, and early clinical response.^11^ In Warm/Wet-R, immediate priorities often include oxygenation, ventilatory support, treatment of the trigger, and afterload reduction when blood pressure allows. Diuretics should not be reflexively withheld, but automatic high-dose escalation may be inappropriate when systemic venous congestion is absent or minimal. Greater caution is warranted in patients with abrupt hypertensive pulmonary oedema, minimal peripheral congestion, low or collapsible venous ultrasound findings, frailty, poor oral intake, borderline blood pressure, or rapid improvement after vasodilator therapy. Conversely, prominent systemic or mixed congestion supports a greater role for decongestive therapy with reassessment of response. Once patients are clinically stable, guideline-directed medical therapy should be initiated or intensified when appropriate.^3,4,12^

Cold-and-dry remains a cautionary profile. It should trigger active exclusion of hypovolaemia, sepsis, pulmonary embolism, tamponade, arrhythmia-related low output, and mechanical complications. Cold-and-wet identifies hypoperfusion with congestion and may overlap with cardiogenic shock; assessment with contemporary shock frameworks is appropriate when hypotension, hypoperfusion, or escalating support is present.^10,13^ Wet-R or Wet-V qualifiers may inform respiratory stabilization and decongestion strategy in cold-and-wet patients, but decisions regarding inotropes or mechanical circulatory support require assessment of shock severity, haemodynamics, trajectory, and response to initial treatment.

This proposal is conceptual and requires prospective validation. Future studies should test whether Wet-R and Wet-V phenotyping is reproducible at the bedside, whether it predicts mortality, rehospitalisation, renal outcomes, or need for advanced support, and whether it changes treatment decisions in a way that improves outcomes.

The aim is not to replace the Nohria–Stevenson framework, but to strengthen its bedside usefulness. Warm-and-dry should remain a diagnostic profile. Within the wet spectrum, distinguishing redistribution-predominant from volume-overload-predominant congestion may improve diagnostic precision, early stabilization, decongestion strategy, and research enrolment, particularly in acute-care settings where rapid decisions often precede comprehensive haemodynamic assessment. **Such bedside phenotyping is complementary to the broader clinical distinction between worsening and advanced heart failure, which carries its own management priorities and challenges.**^14^

## Declarations

### Data Availability

No original patient-level data were generated or analysed for this Viewpoint.
